# Third-trimester persistent maternal hypotension effects on late-onset small for gestational age and adverse perinatal outcomes

**DOI:** 10.1371/journal.pone.0286900

**Published:** 2023-07-14

**Authors:** Mefkure Eraslan Sahin, Erdem Sahin, Mehmet Mete Kirlangic

**Affiliations:** 1 Department of Obstetrics and Gynecology, Kayseri City Hospital, Kayseri, Turkey; 2 Department of Obstetrics and Gynecology, Kartal Dr. Lutfi Kirdar City Hospital, Istanbul, Turkey; Ankara Etlik City Hospital, TURKEY

## Abstract

**Introduction:**

The purpose of the current study was to examine whether third-trimester persistent maternal hypotension is related to small for gestational age (SGA) and adverse perinatal outcomes.

**Methods:**

In this large retrospective cohort study, 6560 pregnant women were enrolled and divided into two groups according to maternal tension status: a normotensive group (n: 6290) and a persistent maternal hypotensive group (n: 270). Persistent maternal hypotension was defined as an SBP <100 mmHg and/or DBP <60 mmHg at three antenatal visits: visit 1 (26^0/7^–29^6/7^ gestation weeks), visit 2 (30^0/7^–33^6/7^ gestation weeks), and visit 3 (34^0/7^–36^6/7^ gestation weeks). Following the Delphi consensus criteria, a fetal growth restriction diagnosis was employed. The presence of an SGA neonate was the primary outcome of the study. SGA was defined as fetal abdominal circumference below the 10th percentile or fetal birth weight below the 10th percentile in the absence of abnormal Doppler findings. The secondary outcomes were defined as the presence of other adverse perinatal outcomes.

**Results:**

The baseline characteristics of the study population were similar. We found that SGA rates were 6.3% in the control group and 7.0% in the persistent maternal hypotension group, which were statistically similar. In the present study, the secondary outcomes which prematurity, low 5-min Apgar score, and NICU admission were similarly between groups.

**Discussion:**

Our results indicate that third-trimester persistent hypotension is not associated with SGA neonates or adverse perinatal outcomes. Hence, it can be concluded that third-trimester persistent hypotension is a physiological phenomenon during pregnancy that should not cause anxiety in mothers.

## Introduction

The term "small for gestational age (SGA)" refers to a condition evident in the fetus at birth that is associated with neonatal morbidity and neonatal death [1]. Despite biochemical evaluations and Doppler ultrasound examinations, it remains challenging for obstetricians to predict SGA early since these measurements are typically standard in uncomplicated pregnancies with SGA neonates [2]. There is substantial evidence in the literature that SGA is associated with adverse neonatal outcomes even in uncomplicated term pregnancies [3–5]. A significant risk of perinatal mortality and morbidity is associated with SGA babies. However, it is possible to greatly minimize these risks if they are identified early by providing immediate neonatal care to the babies and organizing a timely delivery. In light of this, it is of utmost importance to predict SGA and its risk factors early in the process.

Hypertension in pregnancy is a well-explained phenomenon today, but when we look at our routine practice, we encounter maternal hypotension secondary to physiological changes in pregnancy. These hemodynamic changes in the third trimester are caused by a decrease in maternal systolic blood pressure (SBP) and diastolic blood pressure (DBP) [6, 7]. A question frequently asked by pregnant women is: ’Is this persistent hypotension affecting my fetus’s growth or is this situation related to adverse perinatal outcomes?’ This study sought to answer this question considering that maternal hypotension can lead to a chronic decrease in blood flow to the uterus, which leads to subsequent poor fetal growth and placental hypoperfusion [8]. There are limited studies on fetal growth and maternal hypotension in the literature, but those that exist have inconsistencies in the definitions of hypertension and do not represent the contemporary population.

The hypothesis examined was that pregnant women with persistent hypotension might have chronically low placental perfusion, insufficient fetal growth, and a high risk of neonatal SGA compared to those without persistent hypotension. This hypothesis was tested with a large cohort of pregnant subjects. This study evaluated if SGA and adverse perinatal outcomes are caused by persistent maternal hypotension in the third trimester.

## Materials and methods

This retrospective study was approved by the Kayseri City Hospital Ethics Committee (Decision no: 747) and followed the Declaration of Helsinki. The study was conducted at Kayseri City Hospitals in Kayseri, Turkey. Since the study was conducted with retrospective data analysis, an informed consent form was not obtained. Ethics committee waived the requirement for informed consent.

**Fig 1:**
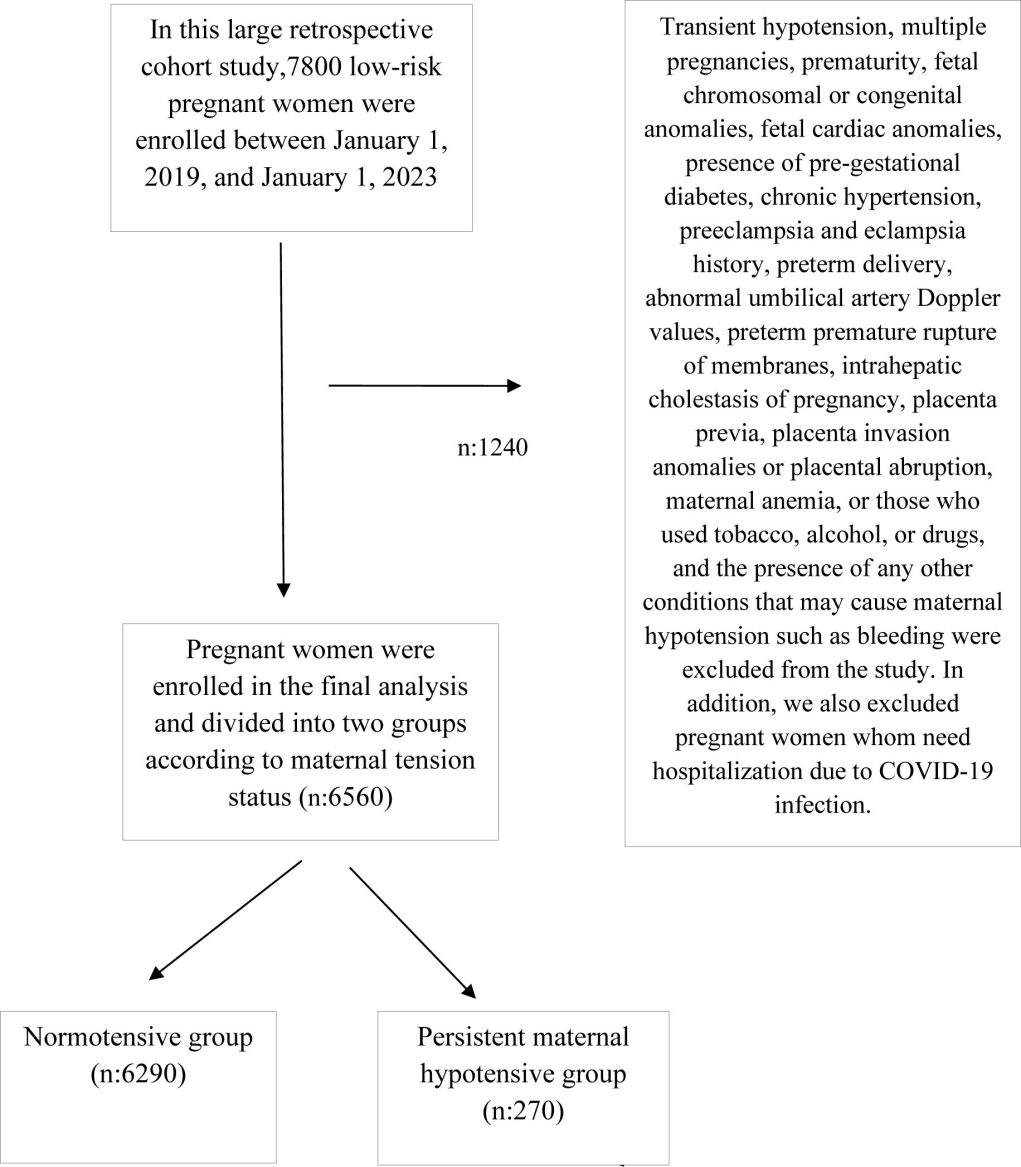
Flow chart of the study population.

This large retrospective cohort study enrolled 7800 low-risk pregnant women between January 1, 2019, and January 1, 2023. The study excluded 1240 of these pregnant women who had multiple pregnancies, prematurity (delivered before 37 weeks), presence of pregestational diabetes or gestational diabetes, chronic hypertension, preeclampsia or eclampsia history, fetal chromosomal or congenital anomalies, abnormal umbilical artery Doppler values, preterm premature rupture of membranes, placental invasion disorders or placental abruption, maternal anemia, or used alcohol or drugs, smoking, or had the presence of any other conditions that may cause maternal hypotension such as bleeding. In this study we also excluded pregnant women whom need hospitalization due to COVID-19 infection. We also included outpatients infected group with mild or no symptoms of infection. In direction of our clinical experience non symptomatic mild COVID-19 infection was not related with SGA and adverse perinatal outcomes. There are comments claiming a relationship between COVID 19 and blood pressure change. Taking blood pressure measurement will affect the results, especially in the presence of active COVID-19 infection. We indicate that we did not receive blood pressure readings in the presence of active COVID-19. Overall, 6560 pregnant women were enrolled in the study and divided into two groups according to maternal tension status: normotensive group (n: 6290) and persistent hypotensive group (n: 270) (Fig 1).

Blood pressure measurements were taken 2 times by resting for at least 20 minutes in a quiet environment. Blood pressure values were recorded in mmHg units. Persistent maternal hypotension was defined as an SBP <100 mmHg and/or DBP <60 mmHg at three antenatal visits: visit 1 (26^0/7^–29^6/7^ gestation weeks), visit 2 (30^0/7^–33^6/7^ gestation weeks), and visit 3 (34^0/7^–36^6/7^ gestation weeks) according to previous studies [8–10]. Transient hypotension was defined as an SBP <100 mmHg and/or DBP <60 mmHg at any 1 of 3 or 2 of 3 these consecutive visits. Pregnant women with transient hypotension were excluded because of the hypotheses that transient hypotension would not causes a poor placental perfusion is a reflection of a chronic process. In addition, we excluded the pregnant women who were followed up but delivered at 37 weeks of gestation before completing their blood pressure measurements at every 3 visits. We specifically chose visits early in the third trimester because it coincides with the beginning of accelerated fetal growth. For instance, the 50th percentile fetal weight is 910 grams at 26 weeks of gestation, while it is 3600 grams at 39 weeks of gestation. The fetal weight increases by four times during this period, and when there is poor placental perfusion, fetal growth retardation can occur due to insufficient circulation and oxygenation in the tissues. The gestational week was determined based on the woman’s last menstrual period. Gestational age reanalyzed dating by ultrasound CRL in 1st trimester at all pregnant women. The Delphi consensus criteria were adopted to diagnose fetal growth restriction [11]. The primary outcome of interest was the delivery of an SGA neonate. In the absence of abnormal Doppler findings, SGA was defined as fetal abdominal circumference below the 10th percentile or fetal birth weight below the 10th percentile [11]. Prematurity was defined as delivery before 37 gestational weeks.

SPSS for Windows version 20.0 (SPSS Inc., Chicago, IL) was employed to carry out statistical analysis. The Shapiro–Wilk test was used to compare two groups to determine the normality of the data, and the Levene’s test was used to test the assumption of homogeneity of variance. The values are expressed as the mean ± standard deviation. Parametric comparisons were made using Student’s t-test, and nonparametric comparisons were made using the Mann–Whitney U test. n% values were analyzed using the Pearson chi-squared test.

## Results

Table 1 shows the study population’s baseline maternal characteristics. The groups had similar maternal age, nulliparity, BMI before delivery (kg/m^2^), ethnicity, and previous Cesarean delivery rates (p = 0.680, p = 0.750, p = 0.910, p = 0.840, and p = 0.320, respectively).

**Table 1:** Comparison of maternal characteristics between groups.

	Control (n = 6290)	Persistent Maternal Hypotension (n = 270)	P value*
Maternal age (years)	28.1 ± 4.7	29.3 ± 4.3	0.680
Nulliparity	2138 (33.9)	83 (30.7)	0.750
BMI before delivery (kg/m^2^)	28.8 ± 3.4	29.1 ± 3.6	0.910
Ethnicity (Caucasian)	5150 (81.8)	224 (83.7)	0.840
Previous CS history	1820 (28.9)	90 (33.3)	0.320
Maternal SBP at first visit (mmHg)	117.4 ± 10.1	90.68 ± 8.4	<0.001
Maternal DBP at first visit (mmHg)	83. 5 ± 5.1	58.4 ± 4.2	<0.001
Maternal SBP at second visit (mmHg)	119 ± 11.3	90.2 ± 7.4	<0.001
Maternal DBP at second visit (mmHg)	84.4 ± 6.1	56. 3 ± 5.3	<0.001
Maternal SBP at third visit (mmHg)	122.4 ± 10.8	90.8 ± 8.6	<0.001
Maternal DBP at third visit (mmHg)	83.3 ± 7.6	58. 2 ± 5.3	<0.001

Notes: BMI, body mass index; CS, Cesarian section; SBP, Systolic Blood pressure; DBP; Diastolic Blood pressure. Values are presented as n (%) or mean ± standard deviation.

*Values of n (%) were compared using the Chi-squared test

Table 2 shows a comparison of the perinatal characteristics and SGA rates between groups. In the control group, the gestational age at delivery was 39 weeks (38–40), whereas in the maternal hypotension group, it was 38 weeks (38–40) (p = 0.210). Both groups had similar delivery induction rates due to delivery doesn’t start spontaneously, male fetal gender, spontaneous vaginal delivery rates and emergency Cesarean section rates (p = 0.620, p = 0.350, p = 0.680 and p = 0.760, respectively). The presence of SGA is the primary outcome of the study. We found that SGA rates were 6.3% in the control group and 7.0% in the persistent maternal hypotension group, which was statistically similar (p = 0.890). Mean birth weight at delivery and birth weight percentile at delivery were statistically similar between groups (p = 0.540 and p = 0.920). The secondary outcomes were prematurity, low 5-min Apgar score, and NICU admission. In the present study, we did not find any significant differences in prematurity, low 5-min Apgar score, or NICU admission rates between groups (p = 0.580, p = 0.840, and p = 0.640, respectively).

**Table 2:** Comparison of perinatal outcomes and small for gestational age rates between groups.

	Control (n = 6290)	Persistent Maternal Hypotension (n = 270)	P value*
Gestational age at delivery (weeks)	39 (38–40)	38 (38–40)	0.210
Prematurity	490 (7.7)	18 (6.6)	0.580
Delivery induction rates due to delivery does not start spontaneously	754 (11.9)	35 (12.9)	0.620
Birth weight (g)	3240 ± 290	3050 ± 260	0.540
Birth weight percentile	60.8 ± 8.4	64.8 ± 8.9	0.920
SGA rates	402 (6.3)	19 (7.0)	0.890
Fetal male gender	3390 (53.8)	151 (55.9)	0.350
Spontaneous vaginal delivery rates	3376 (53.8)	129 (47.9)	0.680
Elective CS rates	1820 (28.9)	90 (33.3)	0.320
Emergency CS rates	340 (5.4)	16 (5.9)	0.760
• Fetal distress	205 (3.25)	10 (3.70)	
• Obstructed labor	110 (1.76)	4 (1.44)	
• Umbilical cord prolapses	9 (0.14)	1 (0.37)	
• Placental abruption	16 (0.25)	1 (0.37)	
Apgar score 5 min<7	22 (0.34)	3 (1.1)	0.840
NICU admission rates	132 (2.0)	8 (2.9)	0.640

Notes: CS, Cesarian section; SGA, small for gestational age. Values are presented as median (25th-75th percentile), mean ± standard deviation, or n (%).

*Values of n (%) were compared using the Chi-squared test

## Discussion

The main aim of the present study was to evaluate the role of third-trimester persistent maternal hypotension on SGA and adverse perinatal outcomes. The key findings are as follows: 1) approximately 6.4% of newborns had SGA and third-trimester persistent maternal hypotension was not associated with SGA neonates; 2) third-trimester persistent maternal hypotension was not a risk factor for prematurity, low 5-min Apgar score, or NICU admission. Hence, it can be concluded that third-trimester persistent hypotension can be a physiological phenomenon during pregnancy.

In the literature, several studies have evaluated the role of maternal hypotension in adverse perinatal outcomes. In the past few decades, evidence linked perinatal complications to low placental perfusion and neonatal mortality [12, 13]. Studies have demonstrated that iatrogenic maternal hypotension treated with atenolol is related to fetal growth and uteroplacental function, further supporting the biological plausibility hypothesis that persistent hypotension is related to fetal growth and uteroplacental function [14, 15]. In a well-designed cohort study published recently, de Los Reyes et al. reported a significant correlation between persistent hypotension and SGA in neonates delivered to low-risk nulliparous mothers [8]. Los Reyes et al.’s paper is the first and largest scale study in the literature that evaluated maternal persistent hypotension effects. In this study, maternal hypotension evaluated in the first and second trimesters and the effect of the presence of transient maternal hypotension on SGA was clearly shown. The most important difference of our study is the evaluation of maternal persistent hypotension from the early third trimester, which is the accelerated fetal development stage [8]. In addition, approximately 85% of our study population consisted of Caucasian ethnicity and the rest were Syrian immigrants.

In the current study, we found that third-trimester persistent maternal hypotension was not associated with SGA neonates or adverse perinatal outcomes such as prematurity, low 5-min Apgar score, and NICU admission. We can explain our results as a physiological phenomenon. It is well documented that peripheral vascular resistance decreases during pregnancy, and this physiological state causes a decrease in SBP and DBP. Additionally, maternal hypotension due to vena cava compression is a physiological condition seen frequently, especially in the third trimester [6, 7]. In routine practice, many pregnant women are concerned that this persistent hypotension may cause fetal growth restriction or other adverse perinatal outcomes. As a result of this study, it is now possible to tell them that this is a physiological condition that does not harm the fetus.

We are aware that our study has some strengths and limitations. The first strength was the size of the study, with more than 6000 pregnant women with complete maternal and neonatal data. In addition, the concentration on the period of accelerated fetal growth in the early third trimester is a most important strength. Los Reyes et al. measured blood pressure during three antenatal visits between gestational weeks 6 0/7 and 29 6/7 for singleton nulliparous pregnancies that delivered at ≥ 24 weeks gestation [8]. A critical difference is that we included a different gestational age range. The next strength of our study was the design. Most past data were from more than 20 years prior to this study, whereas this study provided data from a contemporary cohort. Additionally, one of our study’s most substantial aspects was that our population was a low-risk population. It should be noted that this study did not consider potential confounders that may boost the risk of SGA neonates. On the other hand, there are some limitations to our study. It appears that the retrospective design is a significant limitation. Due to the use of low-risk pregnant women in this study, it is not possible to provide evidence of whether the relationship between adverse perinatal outcomes and persistent hypotension is generalizable to other populations. In addition, long-term follow-up values are not available because we cannot perform continuous monitoring (for example, measurement with Holter). Also, SGA can be affected by a wide variety of factors and we do not know about most of the factors that can affect SGA.

## Conclusions

Our results indicate that third-trimester persistent maternal hypotension is not associated with SGA or adverse perinatal outcomes. Hence, it can be concluded that third-trimester persistent maternal hypotension is a physiological phenomenon during pregnancy, and this should not cause anxiety in mothers.

## References

[1] Obstetricians ACo, Gynecologists. ACOG Practice Bulletin No. 204: fetal growth restriction. Obstetrics and gynecology. 2019;133(2):e97–e109. doi: 10.1097/AOG.0000000000003070 30681542

[2] Oros D, Figueras F, Cruz‐Martinez R, Meler E, Munmany M, Gratacos E. Longitudinal changes in uterine, umbilical and fetal cerebral Doppler indices in late‐onset small‐for‐gestational age fetuses. Ultrasound in obstetrics & gynecology. 2011;37(2):191–5. doi: 10.1002/uog.7738 20617509

[3] Eraslan Sahin M, Sahin E, Col Madendag I, Madendag Y, Acmaz G, Ozdemir F, et al. Evaluation of midtrimester ductus venosus diameter and peak systolic velocity to predict late onset small for gestational age fetuses. The Journal of Maternal-Fetal & Neonatal Medicine. 2022;35(20):3984–90. doi: 10.1080/14767058.2020.1846175 33190543

[4] Sahin ME, Madendag IC, Sahin E, Madendag Y, Acmaz G, Bastug O, et al. Fetal pulmonary artery acceleration/ejection ratio for transient tachypnea of the newborn in uncomplicated term small for gestational age fetuses. European Journal of Obstetrics & Gynecology and Reproductive Biology. 2020;247:116–20. doi: 10.1016/j.ejogrb.2020.02.018 32113059

[5] Col Madendag I, Eraslan Sahin M, Madendag Y, Sahin E, Demir MB, Acmaz B, et al. The effect of iron deficiency anemia early in the third trimester on small for gestational age and birth weight: a retrospective cohort study on iron deficiency anemia and fetal weight. BioMed Research International. 2019;2019. doi: 10.1155/2019/7613868 31886249 PMC6893279

[6] Thornburg KL, Jacobson S-L, Giraud GD, Morton MJ, editors. Hemodynamic changes in pregnancy. Seminars in perinatology; 2000: Elsevier. 10.1016/s0146-0005(00)80047-6 10709851

[7] Ouzounian JG, Elkayam U. Physiologic changes during normal pregnancy and delivery. Cardiology clinics. 2012;30(3):317–29. doi: 10.1016/j.ccl.2012.05.004 22813360

[8] de Los Reyes S, Plunkett BA, Dude A. The association between persistent maternal hypotension and small for gestational age neonates. American Journal of Obstetrics & Gynecology MFM. 2021;3(6):100449. doi: 10.1016/j.ajogmf.2021.100449 34314852

[9] Haas DM, Parker CB, Wing DA, Parry S, Grobman WA, Mercer BM, et al. A description of the methods of the Nulliparous Pregnancy Outcomes Study: monitoring mothers-to-be (nuMoM2b). American journal of obstetrics and gynecology. 2015;212(4):539. e1–.e24. doi: 10.1016/j.ajog.2015.01.019 25648779 PMC4387083

[10] Warland J, McCutcheon H, Baghurst P. Maternal blood pressure in pregnancy and stillbirth: a case-control study of third-trimester stillbirth. American journal of perinatology. 2008;25(05):311–7. doi: 10.1055/s-2008-1075031 18444213

[11] Gordijn S, Beune I, Thilaganathan B, Papageorghiou A, Baschat A, Baker P, et al. Consensus definition of fetal growth restriction: a Delphi procedure. Ultrasound in Obstetrics & Gynecology. 2016;48(3):333–9. doi: 10.1002/uog.15884 26909664

[12] Ng PH, Walters WA. The effects of chronic maternal hypotension during pregnancy. Australian and New Zealand journal of obstetrics and gynaecology. 1992;32(1):14–6. doi: 10.1111/j.1479-828x.1992.tb01888.x 1586326

[13] Friedman EA, Neff RK. Hypertension-hypotension in pregnancy: Correlation with fetal outcome. Jama. 1978;239(21):2249–51. 650804

[14] Lydakis C, Lip GY, Beevers M, Beevers DG. Atenolol and fetal growth in pregnancies complicated by hypertension. American journal of hypertension. 1999;12(6):541–7. doi: 10.1016/s0895-7061(99)00031-x 10371362

[15] Easterling TR, Brateng D, Schmucker B, Brown Z, Millard SP. Prevention of preeclampsia: a randomized trial of atenolol in hyperdynamic patients before onset of hypertension. Obstetrics & Gynecology. 1999;93(5):725–33. doi: 10.1016/s0029-7844(98)00522-5 10912975

